# An Eco-Friendly Supercritical CO_2_ Recovery of Value-Added Extracts from *Olea europaea* Leaves

**DOI:** 10.3390/foods13121836

**Published:** 2024-06-11

**Authors:** Anastasia Kyriakoudi, Ioannis Mourtzinos, Katarzyna Tyśkiewicz, Stoja Milovanovic

**Affiliations:** 1 Laboratory of Food Chemistry and Biochemistry, School of Agriculture, Aristotle University of Thessaloniki (AUTH), 54124 Thessaloniki, Greece; ankyria@agro.auth.gr (A.K.); mourtzinos@agro.auth.gr (I.M.); 2 Łukasiewicz Research Network-New Chemical Syntheses Institute, Al. Tysiąclecia Państwa Polskiego 13a, 24-110 Puławy, Poland; katarzyna.tyskiewicz@ins.lukasiewicz.gov.pl; 3 University of Belgrade, Faculty of Technology and Metallurgy, Karnegijeva 4, 11120 Belgrade, Serbia

**Keywords:** olive leaves, supercritical carbon dioxide extraction, waste valorization, oleuropein, hydroxytyrosol, tyrosol, α-tocopherol

## Abstract

An eco-friendly approach towards the recovery of value-added extracts from olive tree leaves with the aid of supercritical CO_2_ at 30 MPa was carried out. The impact of extraction temperature (35–90 °C) and presence of co-solvents (ethanol, water, and aqueous ethanol) on the total phenolic, flavonoid, and pigment content, as well as oleuropein, hydroxytyrosol, tyrosol, and α-tocopherol content was determined. In addition, the antioxidant activity of extracts from tree leaves using DPPH, ABTS, and CUPRAC assays was investigated. The results of the study showed that the most effective supercritical CO_2_ extraction was at 90 °C with an addition of ethanol, which enabled the separation of extract with the highest content of tested compounds. Some of the highest recorded values were for oleuropein 1.9 mg/g, for carotenoids 5.3 mg/g, and for α-tocopherol 2.0 mg/g. Our results are expected to contribute to the efforts towards the valorization of olive leaves as a sustainable source of valuable compounds, and boost local economies as well as the interest of pharmaceutical, food, and cosmetic industries for novel food by-product applications.

## 1. Introduction

The olive tree (*Olea europaea* L.) is one of the most important and cultivated plants [1,2]. It is usually native to Mediterranean countries such as Spain, Italy, Greece, Algeria, and Tunisia [3]. Olive trees are cultivated for their fruits, which are used for olive oil production as well as for the production of table olives [2,4]. High amounts of by-products, including leaves, are generated annually from olive tree cultivation as well as olive processing [2,5,6]. For instance, olive tree pruning generates waste biomass estimated to vary between 1 and 11 tons per hectare [7] or 25–30 kg per tree [6,8]. Moreover, up to 10% of olives’ total weight arriving at factories are leaves [9,10]. Apart from their main use as animal feed, production of pellets, or for soil amendment, olive leaves have been used in folk medicine to increase energy levels, support the immune system, and treat fever and other diseases [3,8,11]. The use of waste leaves as a source of valuable bioactive compounds could contribute to the agricultural and food sectors that are shifting towards circular and green economies focused on sustainable production with a decrease in resource consumption and waste generation [7,12,13]. It was reported that olive leaves contain numerous bioactive compounds including secoiridoids (e.g., oleuropein, verbascoside), flavones (e.g., apigenin, luteolin, diosmetin), triterpenoids (e.g., oleanolic and maslinic acids), and other phenolic compounds (e.g., hydroxytyrosol, tyrosol, caffeic acid, vanillic acid, ferulic acid) [3,9,14]. Recent articles also suggested olive leaves as a source of extracts for cosmetic products [12] and additives to protect food oil against oxidative damage [15,16].

Considering the numerous health benefits (e.g., antioxidant, anti-inflammatory, hypolipidemic, antimicrobial, cardioprotective, antihypertensive, and anti-carcinogenic) associated with the compounds present in its extracts [1,9,14], the interest of the scientific community towards valorization of olive tree leaves as a cheap, sustainable, and abundant source of bioactive compounds has increased during the last decades [6]. Most of the published studies on the separation of extracts from olive leaves involve the use of conventional techniques, ultrasounds, and microwaves along with conventional solvents (such as water, ethanol, and methanol) as well as novel solvents (such as deep eutectic solvents) [5,8,9,17,18]. However, to the best of our knowledge, the systematic information regarding the use of supercritical fluid extraction (SFE) for the separation of valuable bioactive compounds from olive leaves is limited [6,11]. SFE is an environmentally friendly technique employed for the extraction of bioactive compounds from various plant materials. It is usually performed using a green solvent, namely supercritical carbon dioxide (SC-CO_2_), which has a strong affinity towards non-polar compounds present in plant matrices [10]. Moreover, the extraction conditions can be easily adjusted using polar co-solvents, such as water and ethanol, which are in addition to SC-CO_2_ also generally recognized as safe (GRAS) for use in the food and pharmaceutical industry, to enhance the extraction of target phenolic compounds [1,10]. Canabarro et al. [19] examined the influence of olive leaves’ drying conditions on SFE at 25 MPa and 80 °C and the content of bioactive compounds. Dauber et al. [15] prepared extracts from Arbequina cultivar olive tree leaves with the aid of SC-CO_2_ at 30 MPa and 50 °C with and without ethanol as a modifier to incorporate them in canola oil. Moreover, the production of concentrated oleuropein powder from olive leaves has been reported from alcoholic extracts using SC-CO_2_ assisted extraction at 10–20 MPa and 35–60 °C [14]. However, to the best of our knowledge, to date, there has been no comprehensive study systematically investigating SC-CO_2_ extraction conditions (temperature in the range from 35 to 90 °C and co-solvents water, aqueous ethanol, and ethanol) to identify patterns for optimizing process performance and the yield of targeted valuable compounds (phenolics, flavonoids, pigments, oleuropein, hydroxytyrosol, tyrosol, and α-tocopherol) in extracts from *Olea europaea* tree leaves.

In this view, the objective of the present study was to investigate the SC-CO_2_ extraction conditions, i.e., temperature and presence or absence of a co-solvent on the recovery of olive leaves’ valuable compounds. Moreover, an in-depth characterization of the obtained extracts in terms of yield, total phenolic, flavonoid, oleuropein, hydroxytyrosol, tyrosol, and α-tocopherol content as well as chlorophylls and carotenoids content and in vitro antioxidant activity using a variety of assays was performed. The results of the present study are expected to contribute to the efforts towards olive leaves’ valorization by green technology and to boost by-product utilization for the production of high-added-value natural products.

## 2. Materials and Methods

### 2.1. Plant Material

The olive leaves used in the present study were from the Koroneiki variety (*Olea europaea* var. microcarpa alba) and were collected from the olive grove of the Department of Food Science and Technology of the School of Agriculture of Aristotle University of Thessaloniki at altitude of 39 m during March of 2022. The leaves were dried in an oven at 35 °C for 48 h, homogenized using a Pulverisette 11 Knife Mill (Fritsch GmbH, Idar-Oberstein, Germany) at 6000 rpm for 20 s, sieved using a sieve shaker (type LS10, No. 4082, Retax, Labor Siebmaschine, Hemmingen, Germany) and stored in a freezer (−22 °C) until SFE. The average particle size of grounded olive leaves was <0.55 mm.

### 2.2. Reagents

Carbon dioxide (99.9%) was purchased from Zaklady Azotowe “Puławy” S.A. (Puławy, Poland). Additionally, 3,4,5-Trihydroxybenzoic acid (gallic acid, 97.5–102.5%), rutin (rutin trihydrate, >95.0%), and quercetin (≥95.0% for HPLC) were from Sigma Aldrich (Poznań, Poland), whereas 6-hydroxy-2,5,7,8-tetramethyl-chroman-2-acid (Trolox) (97%) was purchased from Sigma-Aldrich (Stenheim, Germany). Oleuropein, hydroxytyrosol, and tyrosol were from Extrasynthese (Genay, Cedex, France). α-Tocopherol (99%) was purchased from Biosynth (Compton, UK). Sodium carbonate (99.5%, Acros Organics, Geel, Belgium), Folin–Ciocalteau reagent (Sigma Aldrich, Stenheim, Germany), sodium hydroxide (≥98% anhydrous, Sigma Aldrich, Stenheim, Germany), sodium nitrite (pure p.a., Polskie Odczynniki Chemiczne S.A., Gliwice, Poland), aluminum chloride (99.99%, Sigma Aldrich, Stenheim, Germany), diethyl ether (p.a, Chempur, Piekary Śląskie, Poland), and 2,2-diphenyl-1-picrylhydrazyl (DPPH, Sigma-Aldrich, Stenheim, Germany), were used for olive extracts analysis. Moreover, ammonium acetate (CH_3_COONH_4_) (99%), potassium chloride (KCl) (99.5%), sodium chloride (NaCl) (99.8%), sodium dihydrochloride monoacid phosphate (Na_2_HPO_4_•2H_2_O) (99.5%), and potassium dihydrogen phosphate (KH_2_PO_4_) (99.5%) were from Chem-Lab (Zedelgen, Belgium). Copper dichloride dihydrate (CuCl_2_•2H_2_O) (99.99%) was from ThermoFisher (Kandel, Germany), whereas neocuproine (2,9-dimethyl-1, 10-phenanthroline) (≥98%) and the bis-ammonium salt of 2,2′-azino-bis(3-ethylbenzothiazolin-6-sulfonic acid) (ABTS) (≥98%) were purchased from Sigma-Aldrich GmbH (Buchs, Switzerland). Potassium persulfate (K_2_S_2_O_8_) was from Merck (Darmstadt, Germany). HPLC-grade acetonitrile (>99.9%), *n*-hexane (>99.9%), and isopropanol (>99.9%) were from Chem-Lab (Zedelgen, Belgium). Methanol (p.a. min 99.8%) was from Witko (Łódź, Poland) and absolute ethanol from J.T. Baker (Landsmeer, The Netherlands). Ultrahigh-purity water was produced in the laboratory using a Micromatic Wasserlab system (Wasserlab, Spain).

### 2.3. Supercritical Fluid Extraction (SFE) of Olive Leaves

**Figure 1:**
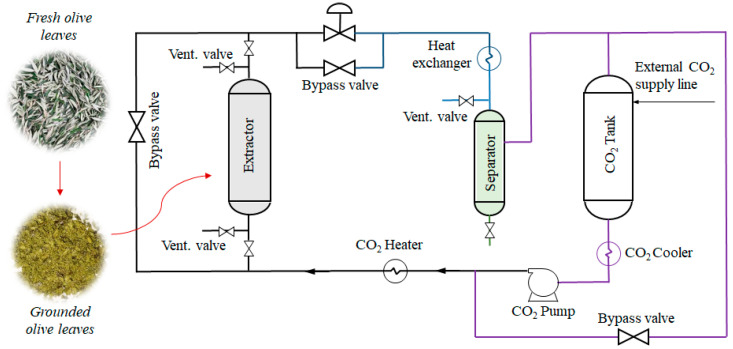
Schematic presentation of the high-pressure unit used for SFE from olive leaves.

Grounded olive leaves (100 g) with a moisture content of 6.54 ± 0.08%, determined using moisture analyzer (MAC 50/1/WH, Radwag^®^, Radom, Poland) prior to SFE, were placed in an extractor vessel of a unit for high-pressure extraction (Figure 1) described in detail elsewhere [20]. The extractor vessel was closed, the temperature and pressure were increased, and the constant flow of SC-CO_2_ (11 kg/h) through the plant material was allowed for 90 min. The mass ratio of solvent (SC-CO_2_) to feed (olive leaves) used was 164 ± 2. The first set of experiments was performed using neat SC-CO_2_ at a constant pressure of 30 MPa and temperatures varying from 35, 50, 70 to 90 °C.

Initial extract analysis indicated that the temperature of 90 °C resulted in the highest yield of total phenolic and flavonoid compounds from olive leaves. Therefore, further SFE processes were performed at 30 MPa and 90 °C using SC-CO_2_ with 50 g of co-solvents. Namely, 100 vol% absolute ethanol, 50 vol% ethanol, or 100% distilled water were poured over the ground olive leaves and allowed to be absorbed. The moistened plant material was then transferred to the high-pressure vessel and exposed to the SFE process. All experiments were performed in duplicate while the extraction yield was calculated as the mass ratio of separated extract (g) and the initial olive leaves material (g) multiplied by 100. After the SFE process, extracts were collected in glass vials and kept in a refrigerator (4 °C) until further analysis. The SFE process conditions as well as the abbreviations of the prepared OLE are presented in Table 1.

### 2.4. Total Phenolic Content in Olive Leaves Extracts

Total phenolic content (TPC) in OLE was estimated by the Folin–Ciocalteau assay. Around 50 mg of each OLE was dissolved in 5 mL methanol. After 15 min of sonication in an ultrasonic bath, 100 μL of each extract solution was mixed with 1.5 mL of distilled water and 100 μL of the Folin reagent. After 5 min, 300 μL of sodium carbonate solution was added. After 40 min in a dark place, all samples were filtered through a syringe filter (450 μm), and the absorbance was measured at 765 nm using a spectrophotometer (V-650, Jasco, Pfungstadt, Germany). Measurements were performed in triplicate and the results were expressed as mg gallic acid equivalents/g dry OLE (mg GAE/g) using an appropriate calibration curve.

### 2.5. Total Flavonoid Content in Olive Leaves Extracts

Total flavonoid content (TFC) in OLE was estimated by the aluminum chloride method as previously described by [21] with some modifications. Around 50 mg of each extract was dissolved in 5 mL methanol. After 15 min in an ultrasonic bath, 0.5 mL extract solutions were mixed with 2 mL distilled water, 0.15 mL sodium nitrite solution (5%), and 0.15 mL aluminum chloride solution (10%). After 5 min, 1 mL sodium hydroxide solution (1 M) was added and a bottle was filled with water to 5 mL. After 30 min in a dark place, all samples were filtered through a syringe filter (450 μm) and absorbance was measured at 510 nm using a spectrophotometer. Measurements were performed in triplicate and the results were expressed as the mass of rutin or quercetin equivalents per dry OLE mass (mg RE/g or mg QE/g) using a calibration curve of rutin or quercetin.

### 2.6. Total Chlorophyll a, Chlorophyll b, and Carotenoid Content in Extracts from Olive Leaves

The content of pigments in OLE was determined as previously described [22,23] with some modifications. The extract samples (28–47 mg) were dissolved in diethyl ether (10 mL). After 10 min in an ultrasonic bath and 10 min in a centrifuge at 7000 rpm, the absorbance of the supernatant was measured at 470, 646, and 662 nm using a spectrophotometer. The content of chlorophyll a, chlorophyll b, and carotenoid in OLE was calculated according Equations (1)–(3). Measurements were performed in triplicate and the results were expressed as the mg of each pigment/g dry OLE.

Chlorophyll a = 10.05 A_662_ − 0.766 A_646_ (1)

Chlorophyll b = 16.37 A_646_ − 3.14 A_662_ (2)

Carotenoids = (1000 A_470_ − 1.28 Chlorophyll a − 56.7 Chlorophyll b)/230 (3)

where A is the absorbance measured at the specific wavelength.

### 2.7. Determination of Oleuropein, Hydroxytyrosol, and Tyrosol Content

Oleuropein, hydroxytyrosol, and tyrosol content of the dry OLE were determined by RP-HPLC-DAD. The HPLC system consisted of an Agilent 1260 Infinity II Quaternary Pump VL, an Agilent 1260 Infinity II Autosampler, and an Agilent 1260 Infinity II Diode Array Detector High Sensitivity. Separation was carried out on an InfinityLab Poroshell 120 EC-C184 μm (150 × 4.6 mm i.d.) column (Agilent Technologies, Santa Clara, CA, USA). Column temperature was set at 30 °C. The mobile phase consisted of water (A) and acetonitrile (B). The elution protocol was based on that described by Martínez-Navarro et al. [24]: 0 min, 5% (B); 10 min, 20% (B); 15 min, 30% (B); 18 min, 30% (B); 36 min, 50% (B); 42 min, 100% (B); 44 min, 100% (B), 48 min, 5% (B); and 49 min, 5% (B). The total run time was 49 min. The flow rate was 1.0 mL/min. The injection volume was 20 µL. Samples prepared using an appropriate amount (50 mg) of dry OLE and 5 mL methanol with the aid of an ultrasonic bath for 15 min were analyzed after filtration through 0.45 μm PTFE filters (Frisenette, Knebel, Denmark). Monitoring was in the range of 190–600 nm. Chromatographic data were processed using the OpenLab CDS version 3.5 software (2021, Agilent Technologies, Santa Clara, CA, USA). Peak identification was based on retention times and spectral characteristics (absorption maxima) with those of available standards. Quantitation of oleuropein, hydroxytyrosol, and tyrosol (mg/100 g dry OLE) was carried out with the aid of calibration curves of properly diluted methanolic solutions of available standards: (i) oleuropein (y = 1.1159x + 12.895, R^2^ =0.999), (ii) hydroxytyrosol (y = 0.5485x + 11.533, R^2^ =0.997), and (iii) tyrosol (y = 0.4359x − 0.9063, R^2^ = 0.999).

### 2.8. Content of α-Tocopherol in Olive Leaves Extract

Determination of α-tocopherol was performed on a LiChrospher-Si column (250 × 4 mm i.d., 5 mm) (MZ Analyzentechnik, Mainz, Germany) according to Psomiadou and Tsimidou [25]. The elution system was isocratic and the mobile phase consisted of n-hexane/2-propanol (99:1, *v*/*v*). The flow rate was 1.1 mL/min and the injection volume was 10 mL. The samples were analyzed after filtration through a 0.45 μm hydrophobic membrane filter. Quantification was carried out using an α-tocopherol calibration curve (y = 3360.8x − 198,948, 40–800 ng/10 µL, R^2^ = 0.99) and fluorescence detection (λexc/λem = 294/330 nm). Samples were analyzed in triplicate.

### 2.9. DPPH Radical Scavenging Activity of Olive Leaves Extracts

DPPH radical scavenging activity of OLE was estimated by the method previously described [26] with some modifications. Extracts (10–50 mg) were dissolved in 5 mL methanol. After 15 min in an ultrasonic bath, dilutions were made. Then, 0.3 mL extract solutions were mixed with 2.7 mL DPPH solution (0.04 mg/mL). After 30 min in a dark place, all samples were filtered through a syringe filter (450 μm) and absorbance was measured at 517 nm using a spectrophotometer. Measurements were performed in triplicate and the results were expressed as IC_50_ (the concentration of an OLE required to scavenge 50% of the initial DPPH radicals).

### 2.10. ABTS Radical Scavenging Activity of Olive Leaves Extracts

The radical scavenging activity of OLE against ABTS radical cation was evaluated according to the protocol of Re et al. [27] and appropriately adjusted. The ABTS solution was prepared by reaction of 5 mL of a 7 mmol/L aqueous ABTS solution and 88 mL of a 140 mmol/L potassium persulfate (K_2_S_2_O_8_) solution. After storage in the dark for 16 h, the radical cation solution was further diluted in PBS (pH 7.4) until an initial absorbance value of 0.70 at 734 nm was attained. An aliquot of each extract (5 µL), after their proper dilution in methanol as described above, was mixed with 2 mL of the ABTS solution. The decrease in absorbance was recorded at 0 and after 6 min. Inhibition of ABTS radical cation in percent (%Inh) was calculated by using the formula %Inh = [Abs734(t = 0) − Abs734(t)] × 100/Abs734(t = 0) after correction with an appropriate blank. These values were converted to Trolox equivalents (μmol TE/100 g dry OLE) via a calibration curve. All of the measurements were performed in triplicate.

### 2.11. Cupric Ion Reducing Antioxidant Capacity (CUPRAC) Assay

The Cu (II) reducing capacity of the extracts was measured according to the protocol of Apak et al. [28]. Briefly, 1 mL of a 0.02 mol/L solution of copper (II) chloride, 1 mL of a 7.5 mmol/L neocuproine solution, and 1 mL of a 1 mol/L ammonium acetate buffer (pH = 7.0) were mixed with 25 µL of the extracts. After the addition of deionized water to a final volume of 4.1 mL, the mixture was shaken for 15 s. The absorbance at 450 nm was measured after the solution had been allowed to stand in the dark for 30 min. The results were finally expressed as Trolox equivalents (μmol TE/100 g dry OLE) after correction with an appropriate blank. All of the measurements were performed in triplicate.

### 2.12. Statistical Analysis

Statistical comparisons of the mean values were performed by one-way ANOVA, followed by the multiple Duncan’s test (*p* < 0.05 confidence level) using the IBM SPSS Statistics for Windows software, Version 27.0 (IBM Corp., Armonk, NY, USA).

## 3. Results and Discussion

### 3.1. Yield of Olive Leaves Extracts Prepared by SFE

**Figure 2:**
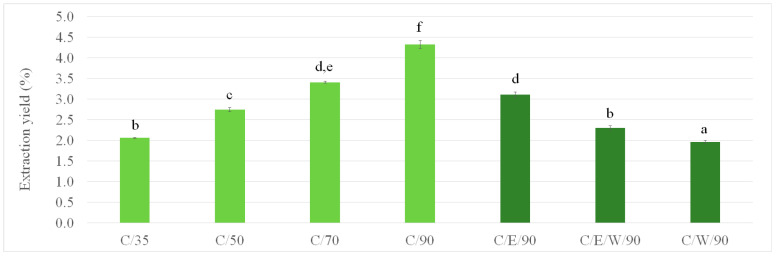
Change in the OLE extraction yield with the change in SC-CO_2_ extraction parameters. Different letters (a–f) suggest that values are significantly different (*p* < 0.05).

In the present study, an environmentally friendly SC-CO_2_ methodology for the extraction of value-added compounds from olive leaves was proposed. Initially, four different extracts were prepared employing temperatures ranging from 35 to 90 °C and neat CO_2_ without any co-solvent. As can be seen in Figure 2, the extraction carried out with neat SC-CO_2_ at 90 °C was found to result in the highest yield (4.3%) followed by SFE at 70 °C (3.4%). A decrease in extraction temperature to 35 °C led to a statistically significant decrease in extraction yield up to 2.1%. The observed increase in separation of OLE with the increase in temperature could be attributed to the vapor pressure enhancement, increased solubility of the bioactive compounds of olive leaves in SC-CO_2_, and facilitated diffusion [10,29,30] despite decreased density of the medium (i.e., SC-CO_2_ density decreased from 929 kg/m^3^ at 35 °C to 704 kg/m^3^ at 90 °C). Previously reported yields of SFE indicated slightly lower values compared to the one obtained in this study. The reported yield of OLE obtained at 30 MPa and 50 °C was 1.0% [15], at 25 MPa and 80 °C was 2.8–3.5% depending on the leaves’ drying temperature [5], at 10–30 MPa and 50 or 100 °C was 0.7–2.1% [10], at 45 MPa and 40 °C was up to 0.6% [30], etc. Observed differences can be ascribed to parameters of the SFE process (pressure, temperature, operating time, solvent flow), type of extraction unit, material storage, moisture, and pretreatment [31], as well as the influence of olive tree variety, cultivation, and harvesting time.

Based on the findings presented in Figure 2, all subsequent experiments with co-solvents (ethanol, water, and 50:50 mixture of ethanol with water) were carried out at 90 °C. The results show that the extraction yield decreases with the addition of co-solvents and with an increase in water content in co-solvents. This decrease is a result of a complex balance between OLE compounds’ solubility and their affinity towards a binary mixture of SC-CO_2_ and co-solvent. The complex balance between SC-CO_2_ and co-solvent ethanol was also previously reported for OLE separation at a constant temperature of 50 or 100 °C with an increase in pressure from 10 to 30 MPa [10]. The highest yield obtained with a co-solvent in the current study was 3.2% for the sample C/E/90 which is higher compared to that previously reported for OLE obtained at 30 MPa and 50 °C using ethanol as a co-solvent (1.9%) [15].

### 3.2. Total Phenolic and Flavonoid Content of Olive Leaves Extracts

Table 2 shows the total phenolic content (TPC) detected in OLE obtained using SC-CO_2_. It can be seen that the increase in the temperature results in an increase in the recovery of TPC in OLE. The same observation was reported for extraction from olive leaves using SC-CO_2_ at 33.4 MPa and 80–120 °C with methanol as a co-solvent [29] as well as using accelerated solvent extraction with aqueous ethanol at 10.3 MPa and temperatures 60 and 80 °C [1]. Moreover, the results of the current study show that the addition of ethanol or aqueous ethanol as a co-solvent significantly increased TPC. The highest TPC of up to 300 mg GAE/g was observed for the SC-CO_2_ extract where ethanol was used as a co-solvent (C/E/90). On the other hand, the use of water as a co-solvent significantly decreased the recovery of TPC. These results are consistent with the fact that solvent polarity is crucial for the extraction of phenolics [15,32]. The use of ethanol as a co-solvent increases the polarity of CO_2_, thus resulting in intensified recovery of polyphenols. Moreover, the increase in phenolic compound separation from leaves might be due to the effect of ethanol on the cell membrane permeability related to the alteration of the phospholipid bilayer [32].

Values of TPC recorded for the supercritical extracts obtained in the current study are higher compared to literature reports on TPC in olive tree leaves extracts. For instance, the highest TPC values of 19.6 mg GAE/g and 142.7 mg GAE/g were reported for OLE obtained by ultrasound-assisted extraction from 60% aqueous ethanol [7] and 70% aqueous ethanol [18], respectively. A value of 26.1 mg GAE/g was reported for OLE obtained at 30 MPa and 50 °C with co-solvent ethanol [15], a value of 113.3 mg GAE/g was reported for OLE obtained at 30 MPa and 50 °C by maceration in 75% ethanolic solution [15], etc. Reported differences can be explained by the selection of extraction technique, its parameters, and the solvent used. Moreover, TPC content in OLE is also significantly influenced by the tree variety (there are around 2000 cultivars spread throughout olive growing regions), age, conditions of cultivation, and harvesting [9,21]. This observation was confirmed by a recent study that showed a range of TPC in OLE from six olive tree cultivars (obtained by conventional and high-pressure extraction with 60% ethanol) to be 88.2–113.4 mg GAE/g [2].

Polyphenols in leaves are different from those in other parts of a plant. The types of flavonoids present on the surface (e.g., in leaf waxes) are usually highly methylated and lack sugar substitution so they should be more easily extracted using SC-CO_2_ [29]. Moreover, the low polarity of some flavonoids, which have high solubility in oils, facilitates their separation from plant leaves during SC-CO_2_ extraction processes. Relatively high values of TFC (compared to the literature [3,4,33]) of up to 178.0 mg QE/g recorded for OLE in the current study support these statements. Table 2 shows that the trend of TFC change is similar to the trend for TPC values. More specifically, TFC significantly increased with an increase in temperature to 90 °C. Similar observations were previously reported for TFC separation from olive leaves using accelerated solvent extraction with aqueous ethanol at 10.3 MPa and temperatures from 60 to 100 °C [1]. A recent study on eight olive tree Tunisian and Algerian varieties showed a range of TFC in OLE (obtained by maceration with ethanol, hexane, and ethyl acetate) to be from 26.4 to 87.6 mg QE/g [3]. TFC detected in ethanolic extract from three tree Brazilian varieties ranged from 3.9 to 5.9 mg QE/g [4], while TFC in OLE obtained by maceration in water was found to range from 0.02 to 0.7 mg QE/g [33].

Moreover, water as a co-solvent proved to be inferior compared to ethanol. The same observation was reported for ultrasound-assisted extraction from olive leaves with water and ethanol as solvents [9]. Various flavonoids expected in OLE are found in their glycosidic form and this form is known not to achieve high solubility in ethanol for extraction. However, as indicated by our study, water is considered to be less preferable for the extraction of flavonoids from olive leaves [9].

### 3.3. Chlorophyll a, Chlorophyll b, and Carotenoid Content of Olive Leaves Extracts

**Figure 3:**
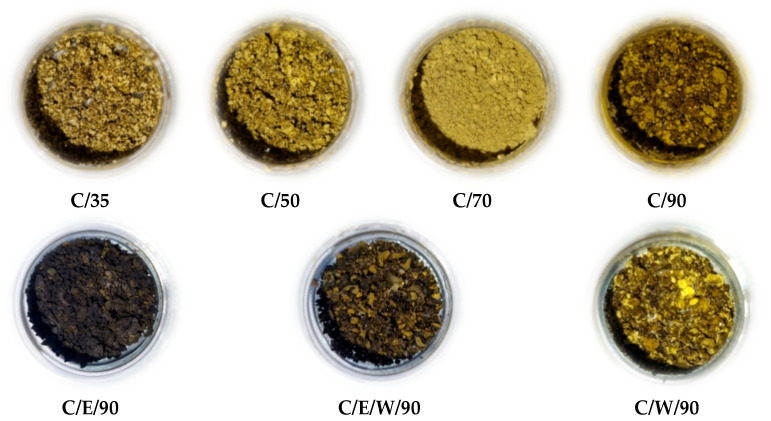
Images of olive leaves extracts obtained using neat SC-CO_2_ (**upper row**) and SC-CO_2_ with a co-solvent (**lower row**).

Changes in process parameters also influenced the color of obtained extracts, as can be seen in Figure 3. The highest temperature of 90 °C used for SFE led to the separation of the darkest color extract. The use of ethanol as a co-solvent to SC-CO_2_ only darkened the color of OLE. The color of extracts is attributed to the presence of pigments that impart color to plants. The appearanceof olive leaves is mainly related to the presence of chlorophylls whose color masks the color of carotenoids that are also present.

The chlorophyll a and chlorophyll b content of the OLE obtained in this study are presented in Table 3. As can be seen, the chlorophyll a content was found to range from 1.8 to 11.7 mg/g dry OLE whereas the chlorophyll b content was found to range from 0.03 to 1.4 mg/g dry OLE. The highest chlorophyll a and chlorophyll b content values were observed for the C/E/90 sample (where ethanol was used as a co-solvent) as expected due to the darkest color seen in Figure 3. To the best of our knowledge, no data exist regarding the chlorophylls’ content of olive extracts obtained with the aid of SC-CO_2_. The content of chlorophylls detected in OLE obtained by conventional extractions is lower compared to values detected in extracts obtained in the current study by SC-CO_2_. For instance, it was reported that chlorophyll a and b, detected in OLE obtained by leaves maceration in acetone, range from 0.5 to 1.1 mg/g and from 0.2 to 0.5 mg/g, respectively [4], total chlorophylls in OLE obtained using 70% ethanol ranged from 0.5 to 0.8 mg/g [16], etc.

Content of chlorophylls is important considering that they can be used in numerous industries, from food to pharmaceuticals, textiles, and cosmetics [34]. For instance, intense green color enables application of chlorophylls for eco-friendly dying of food and textiles [34,35]. Beside intense color, chlorophylls are also known for their antioxidant and anti-inflammatory properties [35]. They can neutralize free radicals, which are unstable molecules that can cause oxidative stress and damage cells. Incorporating chlorophylls into the diet during early life can decrease weight gain and improve glucose tolerance, thus preventing obesity [35]. However, under certain conditions, chlorophylls can exhibit prooxidant behavior. For example, in the presence of light, chlorophylls can generate reactive oxygen species through photodynamic action, which can lead to oxidative damage [4]. This dual role depends on the environmental conditions and the balance between the chlorophyll’s antioxidant defenses and its potential to produce reactive oxygen species.

A similar trend to change in chlorophyll content was observed also for the carotenoids content, which ranged from 2.7 to 5.3 mg/g dry OLE. Olive leaves are expected to contain various carotenoids (including lutein, zeaxanthin, and violaxanthin), although the specific types and concentrations vary depending on the olive tree variety and the stage of maturity, as well as the cultivating growing conditions. Carotenoids are important organic compounds, which provide color to plant leaves, foods, and agriculture products [4]. However, there is a lack of information in available literature on carotenoids content in OLE. Carotenoids content reported for OLE obtained using 70% ethanol in conventional extraction process ranged from 0.03 to 0.04 mg/g [16].

### 3.4. Oleuropein, Hydroxytyrosol, Tyrosol, and α-Tocopherol Content of Olive Leaves Extracts

The results shown in Table 4 reveal the presence of four constitutes characteristic of OLE composition: oleuropein, hydroxytyrosol, tyrosol, and α-tocopherol. Their levels in olive leaves vary depending on the botanical variety of olive trees, maturity stage, climate, and cultivation practices [8,11].

Oleuropein is the major secoiridoid present in olive leaves that has a strong bitter and astringent taste. It is known as an antibacterial, antioxidative, antiviral, antiatherogenic, cardioprotective, and antihypertensive agent [10,11]. In the present study, the levels of oleuropein were found to range from 1.6 to 187.9 mg/100 g dry OLE. The obtained results are in the range of previously reported data for oleuropein content in OLE obtained by SFE. In particular, it was reported that supercritical extract obtained at 25 MPa and 40 °C using co-solvent ethanol contained oleuropein of 0.4 mg/100 g OLE [12], extract obtained at 30 MPa and 100 °C with neat SC-CO_2_ contained oleuropein of 0.19 mg/100 g OLE while using co-solvent ethanol enabled oleuropein content of 715 mg/100 g OLE [10], etc.

Hydroxytyrosol belongs to the phenolic alcohols and has a great effect against cardiovascular diseases and metabolic syndrome. It exhibits neuroprotection, antitumor, and chemo modulation activity, hence being of high interest to the pharmaceutical industry [6,11]. Hydroxytyrosol mainly derives from the hydrolysis of oleuropein under light, high temperatures, or acids/bases [11]. The levels of hydroxytyrosol recorded for the extracts obtained in this study ranged from 16.7 to 248.2 mg/100 g dry OLE. An increase in recovery of hydroxytyrosol with an increase in ethanol content was also previously reported for OLE obtained by intensification of vaporization by decompression to the vacuum technique [32]. Previously reported hydroxytyrosol values for OLE obtained by conventional extraction using ethanol/water/formic acid mixture at room temperature was in the range from 8 to 20 mg/100 g [5], by maceration in ethanol was in the range from 250.7 to 687.5 mg/100 g [3], by microwaves-assisted extraction in 70% ethanol, 80% methanol, and water was in the range from 1.0 to 11.0 mg/100 g [13], etc.

Tyrosol, another phenolic alcohol, is usually present in trace amounts in olive leaves [36]. It was reported as a compound with antioxidant and antibacterial activity [6]. The levels of tyrosol detected in separated extracts ranged from 38.7 to 49.2 mg/100 g dry OLE (Table 4). Previously reported values for OLE obtained by deep eutectic solvents were in the range from 23 to 47 mg/100 g leaves [17], by maceration in ethanol was in the range from 57.7 to 151.7 mg/100 g extract [3], by maceration in acidified methanol was in the range from 0.5 to 1.3 mg/100 g extract [4], by conventional extraction using 70% ethanol was in the range 0.3–1.4 mg/100 g [16], etc.

It was interesting to notice that the temperature of the SFE process had an adverse effect on α-tocopherol content. Namely, an increase in temperature from 35 °C to 50 and 70 °C decreased α-tocopherol content, while a further increase to 90 °C enabled a significant increase in α-tocopherol content up to 197.0 mg/100 g OLE. The same α-tocopherol content decrease was reported for SFE performed at 25 MPa and temperatures 40 and 50 °C [30]. The addition of ethanol to SC-CO_2_ leads to an insignificant increase in the recovery of α-tocopherol. Employment of other co-solvents only decreased α-tocopherol content in OLE. Previous reports in available literature on OLE listed values in the range 1.0–8.2 mg/100 g detected in samples obtained by conventional extraction using 70% ethanol [16]. Content of α-tocopherol is very important considering its role in protection against the detrimental effects of free radicals [16,30].

It should be pointed out that oleuropein, hydroxytyrosol, and tyrosol were not detected in samples of OLE obtained using neat SC-CO_2_ at 35, 50, and 70 °C. Moreover, the highest values of oleuropein and hydroxytyrosol compounds were observed for the OLE obtained using SC-CO_2_ with absolute ethanol as a co-solvent, while the highest content of tyrosol was detected in the C/E/W/90 sample. The use of water as a co-solvent significantly decreased the content of all tested compounds.

Oleuropein, hydroxytyrosol, tyrosol, and α-tocopherol are bioactive compounds that can be added to functional foods and beverages to enhance their health benefits, such as improving heart health, boosting the immune system, and providing anti-inflammatory effects.

### 3.5. Antioxidant Activity of Olive Leaves Extracts

The antioxidant activity of plant extracts is important considering that antioxidants are added to fat-based foods and cosmetics to prevent the formation of off-aromas and toxic compounds as a result of lipid oxidation during storage [10,29]. Plant extracts such as OLE are natural alternatives to synthetic antioxidants as they possess comparable or even higher antioxidant activity [29]. Therefore, the antioxidant activity of OLE, obtained by an environmentally friendly process presented in this study, was examined using three assays (DPPH and ABTS radical scavenging activity as well as cupric ion reducing antioxidant capacity). The obtained results are presented in Table 5 where it can be seen how variations in extraction temperature and addition of co-solvents to SC-CO_2_ extraction process can influence the antioxidant activity of OLE.

In particular, the antioxidant activity detected by the DPPH assay and expressed as IC_50_ ranged from 2.7 to 8.7 mg/mL. It was interesting to notice that antioxidant activity decreased with an increase in temperature from 35 °C to 50 and 70 °C while a further increase in temperature to 90 °C increased the antioxidant activity. The antioxidant activity detected by the ABTS assay ranged from 37.5 to 184.9 μmol TE/g for dry OLE. These results are comparable to those previously reported (163.3 μmol TE/g OLE) for SC-CO_2_ OLE obtained at 30 MPa and 50 °C with co-solvent ethanol [3]. Previous reports in available literature on OLE listed values in the range 69.1–113.8 μmol TE/g detected in samples obtained by conventional extraction using 70% ethanol [16].

The antioxidant activity detected by the CUPRAC assay ranged from 258.1 to 999.2 μmol TE/g for dry OLE following a similar trend to that observed for the ABTS radical scavenging activity. An increase in antioxidant activity of OLE from 120.4 to 341.0 mM TE with an increase in ethanol content based on CUPRAC assay was also previously reported for OLE obtained using intensification of vaporization by decompression during employment of the vacuum technique [32]. The same study found a positive correlation between TPC and the antioxidant activity of OLE, which could also be inferred from our study. Lower activity shown for extracts with inferior TPC and TFC values as expected was discussed by other studies on OLE [9]. Moreover, the highest antioxidant activity was shown by the C/E/90 sample followed by the C/E/W/90 sample. Those samples with higher TPC and TFC as well as pigment, oleuropein, hydroxytyrosol, tyrosol, and α-tocopherol content showed remarkable antioxidant performance. Therefore, the high antioxidant activity found in the extracts of olive leaves can be also attributed to a synergistic effect between the different phenolic compounds present. The same observation was previously reported by comparison of OLE antioxidant activity with the activity of constituents separately [15].

## 4. Conclusions

In the present study, an eco-friendly approach toward the recovery of value-added compounds from olive leaves with the aid of SC-CO_2_ was carried out. The impact of SC-CO_2_ extraction conditions, i.e., temperature and presence of co-solvents on the yield, total phenolic, total flavonoid, oleuropein, hydroxytyrosol, tyrosol, α-tocopherol, chlorophyll a, chlorophyll b, and carotenoids content as well as in vitro antioxidant activity using a variety of assays, was reported for the first time. The results of the present study showed that the most effective SC-CO_2_ extraction conditions were 90 °C and 30 MPa using absolute ethanol as a co-solvent. This set of parameters enabled the separation of the highest content of bioactive compounds of interest in the current study. Presented results are expected to contribute to the efforts towards the valorization of olive leaves as a sustainable source of valuable compounds and to boost local economies as well as the interest of local food industries for novel food applications. This study responds to sustainable waste management and opens up new possibilities for the formulation of novel food additives, cosmetic, nutraceutical, and pharmaceutical products by the utilization of natural alternatives and green extraction technology. However, further research is necessary to evaluate the practical application of the extracts in the food industry.

## Figures and Tables

**Table 1:** The SC-CO_2_ process conditions and abbreviations of the respective OLE.

No	Temperature (°C)	Co-Solvent	Ratio of Solvent and Co-Solvent (g/g)	Abbreviation
1	35	-		C/35
2	50	-	-	C/50
3	70	-	-	C/70
4	90	-	-	C/90
5	90	Ethanol	331 ± 1	C/E/90
6	90	Ethanol/water	333 ± 2	C/E/W/90
7	90	Water	328 ± 2	C/W/90

**Table 2:** Total phenolic and flavonoid content in olive leaves extracts.

Sample	TPC (mg GAE/g Dry OLE) ^1,2^	TFC (mg RE/g Dry OLE) ^1,2^	TFC (mg QE/g Dry OLE) ^1,2^
C/35	65.15 ± 1.71 ^b^	4.77 ± 0.17 ^b^	8.00 ± 0.30 ^b^
C/50	59.19 ± 4.73 ^a^	5.91 ± 0.41 ^a^	9.97 ± 0.74 ^a^
C/70	76.06 ± 0.43 ^c^	9.62 ± 0.16 ^c^	16.70 ± 0.29 ^c^
C/90	171.20 ± 0.05 ^e^	41.25 ± 1.85 ^e^	73.94 ± 3.35 ^e^
C/E/90	299.97 ± 5.43 ^g^	98.72 ± 2.94 ^f^	177.96 ± 5.31 ^f,g^
C/E/W/90	270.07 ± 22.24 ^f^	90.31 ± 3.01 ^f^	162.74 ± 5.44 ^f^
C/W/90	108.49 ± 9.19 ^d^	28.39 ± 0.89 ^d^	50.75 ± 1.61 ^d^

^1^ Different lowercase letters as superscripts within the same column differ significantly according to Duncan’s test at *p* < 0.05. ^2^ Each value is the mean of triplicate determinations ± sd.

**Table 3:** Chlorophyll a, chlorophyll b, and carotenoid content in the obtained OLE.

Sample	Chlorophyll a (mg/g Dry OLE) ^1,2^	Chlorophyll b (mg/g Dry OLE) ^1,2^	Carotenoids (mg/g Dry OLE) ^1,2^
C/35	1.80 ± 0.01 ^a^	0.09 ± 0.00 ^a^	3.63 ± 0.01 ^b^
C/50	2.26 ± 0.02 ^b^	0.03 ± 0.00 ^a^	3.50 ± 0.02 ^b^
C/70	2.01 ± 0.01 ^b^	0.07 ± 0.01 ^a^	2.74 ± 0.01 ^a^
C/90	4.57 ± 0.04 ^c^	0.09 ± 0.00 ^a^	4.22 ± 0.00 ^c^
C/E/90	11.68 ± 0.02 ^e^	1.39 ± 0.01 ^d^	5.30 ± 0.01 ^d^
C/E/W/90	8.05 ± 0.01 ^d^	0.80 ± 0.00 ^c^	4.38 ± 0.00 ^c^
C/W/90	4.01 ± 0.02 ^c^	0.49 ± 0.01 ^b^	3.17 ± 0.00 ^b^

^1^ Different lowercase letters as superscripts within the same column differ significantly according to Duncan’s test at *p* < 0.05. ^2^ Each value is the mean of triplicate determinations ± sd.

**Table 4:** Content of oleuropein, hydroxytyrosol, tyrosol, and α-tocopherol in the OLE.

Sample	Oleuropein (mg/100 g Dry OLE) ^1,2^	Hydroxytyrosol (mg/100 g Dry OLE) ^1,2^	Tyrosol (mg/100 g Dry OLE) ^1,2^	α-Tocopherol (mg/100 g Dry OLE) ^1,2^
C/35	ND	ND	ND	177.8 ± 5.9 ^c^
C/50	ND	ND	ND	139.4 ± 3.7 ^a^
C/70	ND	ND	ND	132.9 ± 4.7 ^a^
C/90	1.57 ± 0.47 ^a^	64.51 ± 1.32 ^c^	46.1 ± 0.1 ^b^	197.0 ± 6.3 ^d^
C/E/90	187.82 ± 4.48 ^d^	248.19 ± 8.55 ^d^	48.7 ± 0.4 ^b,c^	199.9 ± 6.9 ^d^
C/E/W/90	35.85 ± 5.58 ^c^	31.36 ± 2.20 ^b^	49.2 ± 0.8 ^b,c^	170.7 ± 5.9 ^c^
C/W/90	9.38 ± 0.85 ^b^	16.68 ± 0.67 ^a^	38.7 ± 3.0 ^a^	149.9 ± 5.3 ^b^

^1^ Different lowercase letters as superscripts within the same column differ significantly according to Duncan’s test at *p* < 0.05. ^2^ Each value is the mean of triplicate determinations ± sd. ND—Not detected.

**Table 5:** Antioxidant activity of OLE produced by the SC-CO_2_ extraction process.

Sample	DPPH (IC_50_, mg/mL) ^1,2^	ABTS (μmol TE/100 g Dry OLE) ^1,2^	CUPRAC (μmol TE/100 g Dry OLE) ^1,2^
C/35	7.73 ± 0.53 ^c^	4752 ± 413 ^b^	31,740 ± 776 ^c^
C/50	8.74 ± 0.59 ^d^	4097 ± 71 ^a,b^	25,811 ± 842 ^b^
C/70	8.64 ± 0.60 ^d^	3747 ± 347 ^a^	18,968 ± 632 ^a^
C/90	3.92 ± 0.08 ^b^	8487 ± 1366 ^d^	72,021 ± 1654 ^d^
C/E/90	2.68 ± 0.52 ^a^	18,493 ± 318 ^f^	99,916 ± 2552 ^f^
C/E/W/90	2.99 ± 0.29 ^a^	13,043 ± 1159 ^e^	96,161 ± 1211 ^f^
C/W/90	7.97 ± 0.81 ^c^	6057 ± 341 ^c^	89,214 ± 2540 ^e^

^1^ Different lowercase letters as superscripts within the same column differ significantly according to Duncan’s test at *p* < 0.05. ^2^ Each value is the mean of triplicate determinations ± sd.

## Data Availability

The original contributions presented in the study are included in the article, further inquiries can be directed to the corresponding author.
